# Characteristics of Children and Adolescents with High Functioning Autism and Optimal Outcome

**DOI:** 10.1192/j.eurpsy.2025.651

**Published:** 2025-08-26

**Authors:** Z. Ayaslan, Ç. Ermiş, H. B. Baykara

**Affiliations:** 1 Department of Child and Adolescent Psychiatry, SBU Dr. Behcet Uz Pediatrıc Diseases And Surgery Trainıng And Research Hospital, Izmir, Türkiye; 2 Department of Child and Adolescent Psychiatry, Queen Silvia Children’s Hospital, Gothenburg, Sweden; 3 Department of Child and Adolescent Psychiatry, Dokuz Eylul University, Izmir, Türkiye

## Abstract

**Introduction:**

In general, Autism spectrum disorders (ASDs) are considered lifelong disorders but recent data suggests that after treatment, symptomatic improvement and even loss of diagnosis can be achieved in some cases. Although there is not yet a consensus, the term ‘optimal outcome’ is generally used for this group of children. Literature on optimal outcome contributes in evaluating treatment efficacy and in identifying factors influencing good prognosis.

**Objectives:**

The aim of this study is to describe a group of children who achieved optimal outcome and compare sociodemographic and clinical features with the cases still being followed up with the diagnosis of High Functioning Autism (HFA).

**Methods:**

This study consists of 60 cases aged 4-18 years who were diagnosed with Autism Spectrum Disorder according to DSM-IV (before 2013) or DSM-5 criteria by clinicians in Dokuz Eylul University Faculty of Medinice Hospital Child and Adolescent Psychiatry Department Outpatient Clinic and during follow-up considered optimal outcome and cases who still meet the diagnosis of High Functioning Autism. The necessary data were collected through retrospective examination of the medical records and during clinical interviews. Comorbid psychopathologies were assessed with the Schedule for Affective Disorders and Schizophrenia for School-Age Children-Present and Lifetime version (K-SADS-PL) semi-structured interview. Childhood Autism Rating Scale (CARS) were applied to evaluate the severity of the symptoms. Intelligence and developmental test results were recorded if available in the medical records for cognitive assessment.

**Results:**

The optimal outcome (OO) group was diagnosed and started special education at a significantly earlier age than the HFA group (p=0.001). The duration of pre-school education was also significantly higher in the optimal outcome group (p=0,023). Symptom severity assessed by CARS at both the time of diagnosis and the current situation was significantly lower in the optimal outcome group (p<0.001, p<0.001). There was no significant difference between the two groups in terms of early verbalization skills and WISC-R scores.

**Conclusions:**

In our study we defined a group of children who lost their diagnosis of autism after special education. Early diagnosis and initiation of special education and less severe ASD symptoms at the time of diagnosis were found to be important factors contributing to optimal outcome.

**Disclosure of Interest:**

None Declared

